# The Telomerase RNA Protein TERP Exerts a New Function in Safeguarding Female Gamete Quality

**DOI:** 10.3390/biomedicines13092166

**Published:** 2025-09-05

**Authors:** Denis A. Nikishin, Maria D. Tkachenko, Elizaveta G. Fofanova, Oleg A. Permyakov, Olga A. Averina, Maria P. Rubtsova

**Affiliations:** 1 Biological Faculty, M.V. Lomonosov Moscow State University, Moscow 119234, Russia; lizchenbio@mail.ru; 2 N. K. Koltzov Institute of Developmental Biology, Russian Academy of Sciences, Moscow 119334, Russia; tkmadm@yandex.ru; 3 Chemistry Faculty, M.V. Lomonosov Moscow State University, Moscow 119234, Russia; norad_m@mail.ru (O.A.P.); averina.olga.msu@gmail.com (O.A.A.)

**Keywords:** TERP, oocyte quality, autophagy, female infertility, meiotic spindle, non-canonical function, lysosome, ovarian reserve

## Abstract

**Objectives:** Oocyte quality is crucial for female fertility, but the underlying molecular mechanisms remain unclear. This study investigates the non-canonical role of the telomerase RNA protein (TERP), whose function in oogenesis is unknown, in safeguarding female gamete quality. **Methods:** We used gain-of-function (AT) and loss-of-function (D7) mutant mouse lines to assess oocyte quality via morphological and molecular analyses. Key methods included immunofluorescence of meiotic spindles, Western blotting for the autophagy marker LC3B, and qRT-PCR to quantify the perinatal ovarian reserve. **Results:** Both AT and D7 mutations caused severe meiotic spindle abnormalities, including aberrant morphology and increased size. The D7 mutation, in particular, led to impaired cytoplasmic maturation and reduced autophagy levels in oocytes. Furthermore, loss of TERP function resulted in an abnormally large ovarian reserve in newborn females, which correlated with decreased expression of autophagy and lysosomal markers in the newborn ovary. **Conclusions:** This study establishes a novel, non-canonical function for TERP as a crucial regulator of oocyte quality. TERP dysregulation compromises meiotic integrity and oocyte maturation by disrupting lysosome-dependent autophagy.

## 1. Introduction

Successful female reproduction depends on the production of a high-quality oocyte through a complex and meticulously orchestrated process. Oocyte quality itself is determined by two major factors: meiotic fidelity and cytoplasmic competence. The accurate segregation of chromosomes during meiosis is paramount, as errors frequently lead to aneuploidy—a leading cause of female infertility, spontaneous miscarriages, and congenital disorders [1,2]. Equally important is the oocyte’s cytoplasmic competence, which relies on the integrity of its organelles and the precise regulation of its cellular machinery [3].

Cellular quality control pathways, particularly autophagy, are essential for remodeling the oocyte cytoplasm during maturation [4]. This catabolic process degrades and recycles damaged organelles and protein aggregates, thereby maintaining cellular homeostasis and supplying the maturing oocyte with essential biomolecules [5]. Consequently, impairments in autophagy are directly linked to compromised oocyte quality and reduced developmental potential [6].

Telomerase is an enzyme best known for its canonical role in maintaining the length of telomeres at the ends of chromosomes, a function critical for protecting genomic integrity [7]. This complex includes a reverse transcriptase (TERT) and an RNA component (TERC). However, beyond this well-established function, components of the telomerase complex possess non-canonical, or “moonlighting,” functions independent of telomere elongation. For example, the catalytic subunit TERT has been shown to modulate the expression of multiple genes, including those involved in cell proliferation, independent of its role in telomere elongation [8,9].

Recently, a small protein named TERP (Telomerase RNA Protein) was identified, encoded within the TERC transcript [10]. The molecular weight of full-length hTERP is 13 kDa, pI–11,7. The predicted molecular weight of mTERC is 15 kDa, pI–11,3. The non-redundant BLAST (https://blast.ncbi.nlm.nih.gov/) searching revealed the 41% identity of hTERP to the predicted protein by the conceptual translation approach protein at *Mus musculis* that is encoded by the mTERC. Putative proteins encoded by telomerase RNA genes of *Fellus catus*, *Equus cabballus* and *Mus musculus* homologous to hTERP were predicted and aligned. The aligned sequences have shown 40 or a higher percentage of identity [10]. While TERP’s role at the organismal level has not been investigated, crucial initial studies have provided insights into its cellular functions in non-reproductive contexts. Previous work in cell lines has implicated human TERP (hTERP) in the regulation of apoptosis and autophagy [10,11]. Notably, TERP deficiency was shown to influence AMPK activity, a key regulator of the mTORC pathway and cellular metabolism [11]. Despite these findings in somatic cells, the physiological role of this peptide, particularly within the highly specialized context of oocyte meiosis and maturation, remains unexplored.

In this study, we investigated the non-canonical role of TERP in mammalian oogenesis using genetically modified mouse lines with gain-of-function (AT) and loss-of-function (D7) mutations. Our principal aim was to determine if TERP expression is critical for female gamete quality. Our results demonstrate that both increased and decreased TERP activity lead to profound defects in meiotic spindle architecture, impaired cytoplasmic maturation, and an altered perinatal ovarian reserve. Crucially, we present evidence linking these diverse phenotypes to the disruption of cellular autophagy. These findings uncover a novel function for the TERC/TERP axis in safeguarding the quality of the female gamete.

## 2. Materials and Methods

### 2.1. Generation and Maintenance of Mutant Mouse Lines

Genetically modified mouse lines were generated by microinjecting F1 hybrid (C57Bl/6J × CBA) zygotes with a mix containing sgRNA (12 ng/μL), *S. pyogenesis Cas9* mRNA (25 ng/μL), and a single-stranded DNA (ssDNA) template (5 ng/μL), as previously described [12]. The sgRNA, with the guiding sequence ACCCTGATTTTCATT-AGCTG, was produced by in vitro transcription from a PCR-amplified fragment of the pX458 plasmid [13]. The sequence of the ssDNA template is provided in the Supplementary Materials.

Mice were sourced from the Federal Research Center Institute of Cytology and Genetics (ICG SB RAS, Novosibirsk, Russia) and housed in individually ventilated cages under controlled conditions (12/12 light cycle; 20–24 °C; 30–70% humidity) with ad libitum access to chow and water.

Founder mice and their progeny were genotyped by PCR amplification and sequencing of tail DNA using the primers CAAATGGGGAAGAGGGAGCA and CCTGCGCTGACGTTTGTTTT. For this study, we used lines with a C23A substitution (AT) and a Δ29-35 deletion (D7). Homozygous mutant mice and wild-type (WT) littermates were obtained by crossing heterozygous carriers. Subsequent experiments compared the two mutant lines (AT and D7) with WT mice, which served as the control group.

The experimental unit was an individual mouse or oocytes from one mouse, as specified in the figure legends. Sample sizes were determined based on preliminary studies to ensure sufficient statistical power while minimizing animal use. A total of 274 animals were used. No data points were excluded unless a sample was damaged during processing. Group allocation was predetermined by genotype, so randomization was not applicable. To minimize bias, experimenters involved in data collection and analysis were blinded to the genotypes. This study was designed and reported in accordance with the ARRIVE guidelines [14]. All animal procedures were performed to minimize stress and were conducted in accordance with the Council of the European Communities Directive of 24 November 1986 (86/609/EEC).

### 2.2. Oocyte Collection

Mature female mice (2 months old) were superovulated via intraperitoneal injection of inhibin antiserum (0.2 mg) and 4 IU of eCG (CARD HyperOva^®^, Cosmobio Ltd., Tokyo, Japan). For GV-oocyte isolation, ovaries were harvested 36 h post-superovulation, rinsed in ice-cold dPBS, and placed in pre-warmed (37 °C) Hanks’ Balanced Salt Solution (HBSS). GV-stage oocytes were released by puncturing antral follicles with a 29G needle. For MII-oocyte isolation, mice received an intraperitoneal injection of human chorionic gonadotropin (hCG, 8 IU) 46 h after the initial superovulation. Oviducts were dissected 16 h post-hCG injection and placed in ice-cold dPBS. Cumulus-oocyte complexes (COCs) were collected from the ampulla using a denudation pipette and transferred to a four-well plate containing HBSS supplemented with 80 U/mL hyaluronidase. After 15 min of incubation to remove cumulus cells, the denuded MII-stage oocytes were washed three times for 5 min each in HBSS.

### 2.3. Immunofluorescence and Confocal Microscopy

Isolated oocytes were fixed with 4% paraformaldehyde in PBS, permeabilized with 0.1% Triton X-100 in PBS (PBST), and treated with 0.5% SDS to remove the zona pellucida. After blocking with 5% FBS in PBST, samples were incubated for 1 h at room temperature with a primary mouse anti-β-tubulin antibody (1:10,000). After washing, samples were stained with a CF 568-conjugated goat anti-mouse IgG secondary antibody (1:500). DNA was counterstained with DAPI to visualize GV chromatin.

Ovaries were fixed overnight in 4% paraformaldehyde, cryoprotected in sucrose solutions (15% then 30%), and embedded in O.C.T. compound. 20-μm cryosections were prepared using a CM1900 cryostat (Leica Biosystems, Deer Park, IL, USA). Sections were permeabilized, blocked, and incubated for 1 h with primary antibodies: rabbit anti-LC3B (1:1000) or rat anti-LAMP1 (1:1000). Samples were then stained with corresponding secondary antibodies: CF 568-conjugated goat anti-rabbit IgG (1:500) or CF 633-conjugated donkey anti-rat IgG (1:500). Sections were also counterstained with FITC-conjugated Lens Culinaris Agglutinin (FITC-LCA, 1:1000). A detailed list of all antibodies and key reagents is provided in Supplementary Table S1.

All samples were mounted in Mowiol and analyzed using a LSM880 laser scanning confocal microscope (Carl Zeiss AG, Oberkochen, Germany) with consistent settings across all groups. Images were processed using FIJI software (ImageJ 2.9.0/1.54f, open source, available at https://imagej.net/software/fiji/ (URL accessed on 19 March 2025)). Spindle size was measured using the area tool. Immunoreactivity intensity was measured as the Mean Gray Value from five Regions of Interest (ROIs) per section in primordial follicle clusters.

### 2.4. Western Blotting

GV oocyte samples (100–200 oocytes per sample) were lysed in RIPA buffer supplemented with a protease/phosphatase inhibitor cocktail. Lysates were denatured in Laemmli buffer for 30 min at 37 °C. SDS-PAGE and protein transfer were performed as previously described [15]. A primary rabbit anti-LC3B antibody (1:1000) was used, followed by a horseradish peroxidase-conjugated goat anti-rabbit IgG secondary antibody (1:50,000). After washing in TBST, protein bands were visualized using an in-house enhanced chemiluminescence (ECL) solution. Band intensities were quantified using Image Lab software (Version 6.0.1, Bio-Rad Laboratories, Inc., Hercules, CA, USA). A detailed list of antibodies and key reagents is provided in Supplementary Table S1.

### 2.5. Quantitative Reverse Transcription-Polymerase Chain Reaction (qRT-PCR)

Total RNA was isolated from ovaries or cells using an RNA extraction reagent (Evrogen, Moscow, Russia). For ovarian reserve assessment, 30 µL of an external RNA standard (in vitro-transcribed *GFP* RNA, 20 ng/mL) was added to each ovarian sample during extraction. Following DNase I treatment, 1 μg of total RNA was reverse transcribed using an M-MLV reverse transcription kit (Evrogen, Moscow, Russia) with random hexadeoxynucleotides.

Quantitative real-time PCR was performed using a StepOnePlus Real-Time PCR System and a SYBR Green-based qPCR master mix (Evrogen, Moscow, Russia). Gene expression levels were normalized to reference genes using the 2^−ΔCt^ method. The ribosomal protein gene *Rps18* and TATA-box binding protein gene *Tbp* served as reference genes due to their stable expression in ovarian tissue [15]. For ovarian reserve assessment, normalization was performed against the external *GFP* standard. Reaction specificity was confirmed by melting curve analysis (Table 1).

To quantify telomeric DNA, primers specific to telomeric repeats were used [16], with the single-copy gene *Rplp0* (*36B4*) serving as an internal control for normalization. This qPCR was performed on genomic DNA extracted from tails.

### 2.6. Statistical Analysis

Sample sizes were selected based on our extensive experience and standards established in previous, similar studies [17,18,19], which have demonstrated that the chosen group sizes are adequate to detect biologically significant differences while adhering to the 3Rs principles of animal welfare. Statistical analyses were performed using GraphPad Prism 9.0 software (GraphPad Software, San Diego, CA, USA). A *p*-value of less than 0.05 (*p* < 0.05) was considered statistically significant. Data are presented as mean ± standard error of the mean (SEM) unless otherwise stated. Prior to conducting parametric statistical tests, all data were assessed for normality of distribution using the Shapiro–Wilk test. If the data for all groups being compared met the assumption of normality, a Student’s t-test was used for pairwise comparisons, and a one-way ANOVA was used for multiple group comparisons, with appropriate post hoc tests (e.g., Tukey’s multiple comparisons test) applied where ANOVA indicated a significant difference. If any of the groups failed the Shapiro–Wilk test for normality, non-parametric tests were employed. Specifically, the Mann–Whitney U test was used for pairwise comparisons, and the Kruskal–Wallis test was used for multiple group comparisons, with Dunn’s multiple comparisons test used for post hoc analysis where the Kruskal–Wallis test indicated a significant difference.

## 3. Results

### 3.1. Description of Genetically Modified Mice

Previously, we demonstrated that the precursor of human telomerase RNA (hTERC) encodes a protein designated as TERP (Telomerase RNA Protein) [10]. This protein has been shown to protect cells from apoptosis and to regulate autophagy by modulating the activity of AMPK and TSC2 kinases [11]. Bioinformatic analyses of TERC genes across various organisms have revealed the presence of homologous open reading frames (ORFs) in a majority of the analyzed species [10]. Notably, the mouse TERP primary structure shares 41% identity with its human counterpart, suggesting a conserved function across species.

The presence of the TERP coding region within the TERC precursor suggests a potential dual function for this RNA species. It is hypothesized that TERC may serve two distinct roles: first, as a component of the telomerase complex in the nucleus for telomere elongation, and second, as a template for TERP synthesis in the cytoplasm [20,21].

The tertiary structure of TR includes a pseudoknot region, which is essential for the assembly and activity of the telomerase complex [22,23]. Notably, the start AUG codon for human TERP translation resides within this pseudoknot, a positioning that may inhibit protein synthesis due to the structural constraints imposed by the RNA. In contrast, the ORF for mouse TERP initiates from a CUG codon, located near the 5′-end of TERC (22 nt). This unconventional start codon may have evolved to facilitate translation with low efficiency. While the structural organization of human TERC may inhibit translation efficacy, the substitution of the canonical AUG start codon with CUG in mouse TERC could similarly impact translational efficiency, albeit in a different manner.

**Figure 1:**
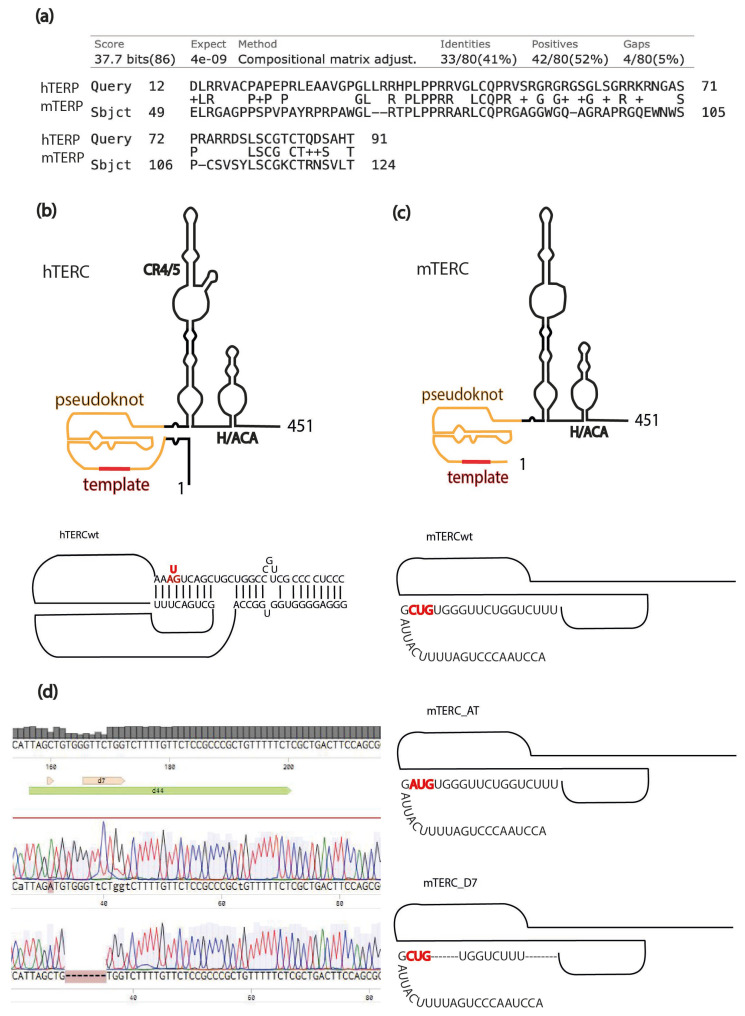
Characterization of mouse telomerase RNA protein (TERP) and mutations in the mouse telomerase RNA component (TERC). (**a**) Amino acid sequence alignment of human (hTERP) and mouse (mTERP) telomerase RNA proteins, showing 41% identity. (**b**) Schematic of the human TERC (hTERC), which contains an AUG start codon (red) for TERP translation. (**c**) Schematics of wild-type (WT) mTERC and the engineered mTERC_AT and mTERC_D7 mutants. The native CUG codon in WT and mTERC_D7 is shown in red. The mTERC_AT mutant contains a CUG-to-AUG conversion (red). (**d**) Sanger sequencing verification of genomic edits. Top: WT reference sequence showing the position of the mutations. Middle: Chromatogram from an mTERC_AT mutant confirming the point mutation. Bottom: Chromatogram from an mTERC_D7 mutant confirming the deletion.

**Figure 2:**
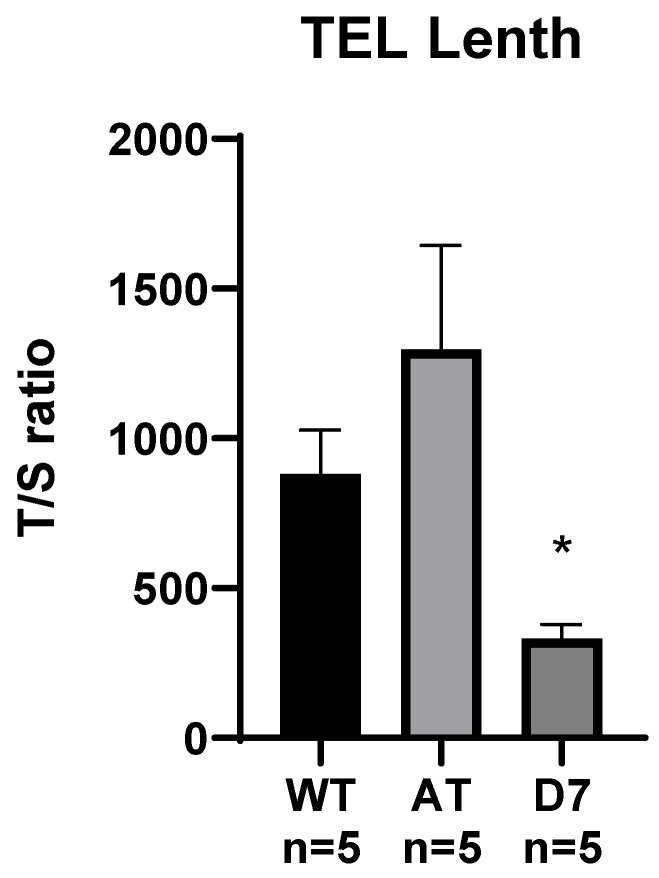
Telomere length analysis in the 5th generation of wild-type and mutant mice via real-time PCR on tail-extracted genomic DNA. Telomere repeats (T/S ratio) normalized to the single-copy gene *Rplp0* using the dCt method. M ± SEM, n—the number of animals analyzed per group; * *p* < 0.05 (Kruskal–Wallis test with Dunn’s multiple comparisons test).

Recently, we demonstrated that the deletion of five nucleotides, including the *TERP* AUG codon, from the human TERC does not affect the functionality of the telomerase complex [24]. To explore the physiological role of TERP in mice, we generated two mouse lines for loss-of-function and gain-of-function studies. Specifically, the deletion of seven nucleotides at positions 29-35 of the TERC gene (D7 line) is expected to result in the absence of TERP, resulting in a loss-of-function genotype, while the substitution of C23 with A in the start codon of the TERP ORF (AT line) is anticipated to enhance TERP translation, yielding a gain-of-function genotype (Figure 1). To assess whether the engineered mutations affected telomerase function, we measured telomere length in 5th-generation mice (Figure 2). This analysis revealed that telomeres were significantly shorter in D7 mutants compared to wild-type animals.

To provide a broader context for our molecular and cellular analyses, we monitored the general health and reproductive performance of the mouse lines (Supplementary Table S2). A cohort of females (n = 6 per line) was observed for 12 months, during which no overt signs of malformations, pathologies, or distress were noted in any group. One female from the D7 line died at 11 months of age from an undetermined cause. Breeding trials revealed that D7 mutant females had reduced fecundity (Supplementary Table S3), with an average litter size approximately 29% smaller than that of wild-type (WT) controls. In contrast, litter sizes for the AT gain-of-function line were comparable to WT. The sex ratio of the pups was not significantly different across the genotypes, and males from all lines exhibited normal fertility.

In summary, the AT line represents a gain-of-function allele, whereas the D7 line represents a loss-of-function allele, providing us with a model to assess the effect of both increased and decreased TERP activity during oogenesis.

### 3.2. Morphological Evaluation of Mature MII Oocytes

**Figure 3:**
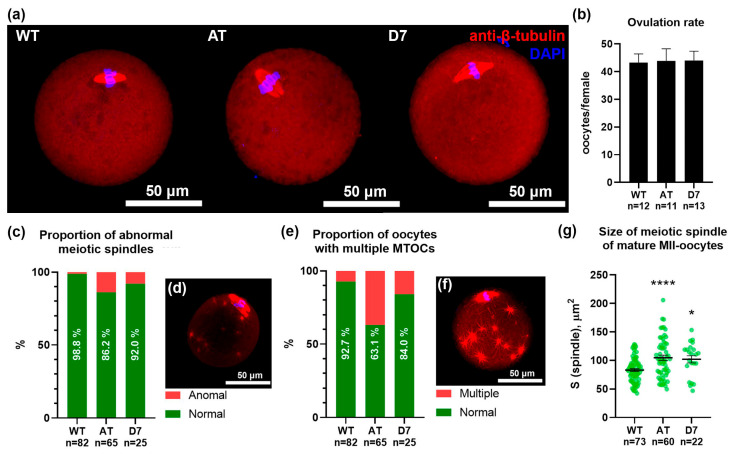
Analysis of MII-oocytes from control (WT) and mutant (AT and D7) female mice. (**a**) MII-oocytes were immunostained with antibodies against β-tubulin to reveal the meiotic spindle for morphologic evaluation. (**b**) Number of ovulated MII-oocytes obtained from oviducts of females induced by HyperOva^®^. n—the number of animals analyzed per group. (**c**) Morphological evaluation of the meiotic spindle in MII-oocytes. n—the number of oocytes analyzed per group. (**d**) MII-oocyte with anomal meiotic spindle. (**e**) Assessment of the tubulin cytoskeleton state in MII-oocytes. n—the number of oocytes analyzed per group. (**f**) MII-oocyte with multiple cytoplasmic microtubule-organizing centers (MTOCs). (**g**) Quantitative analysis of the lateral projection size of the meiotic spindle in MII-oocytes, n—the number of oocytes analyzed per group; * *p* < 0.05, **** *p* < 0.0001 according to Ordinary One-way ANOVA with Tukey’s multiple comparisons test.

To assess the impact of mTERP mutations on oocyte quality, we analyzed MII oocytes from mutant and wild-type (WT) mice (Figure 3). First, we quantified the number of ovulated oocytes after hormonal superovulation, which did not differ significantly between groups (averaging ~45 oocytes/female across all groups; Figure 3b). While initial inspection revealed no gross morphological alterations, detailed confocal microscopy of the meiotic spindle (Figure 3a) uncovered significant structural defects.

Analysis of the confocal images revealed several defects in oocytes from mutant mice. The frequency of aberrant spindle morphologies was significantly higher in both mutant lines (13.8% for AT and 8.0% for D7) compared to WT oocytes (1.2%; Figure 3c). Similarly, the percentage of oocytes containing multiple cytoplasmic microtubule-organizing centers (MTOCs) was elevated in the AT (36.9%) and D7 (16.0%) groups versus the WT group (7.3%; Figure 3d). Finally, the meiotic spindles were significantly larger in oocytes from both mutant lines (104.6 μm^2^ for AT and 101.8 μm^2^ for D7) compared to controls (83.0 μm^2^; Figure 3g).

These results demonstrate that mTERP expression leads to significant abnormalities in meiotic spindle organization and size, indicating a decline in oocyte quality.

### 3.3. Morphological Evaluation of GV-Oocyte Maturation Stage

**Figure 4:**
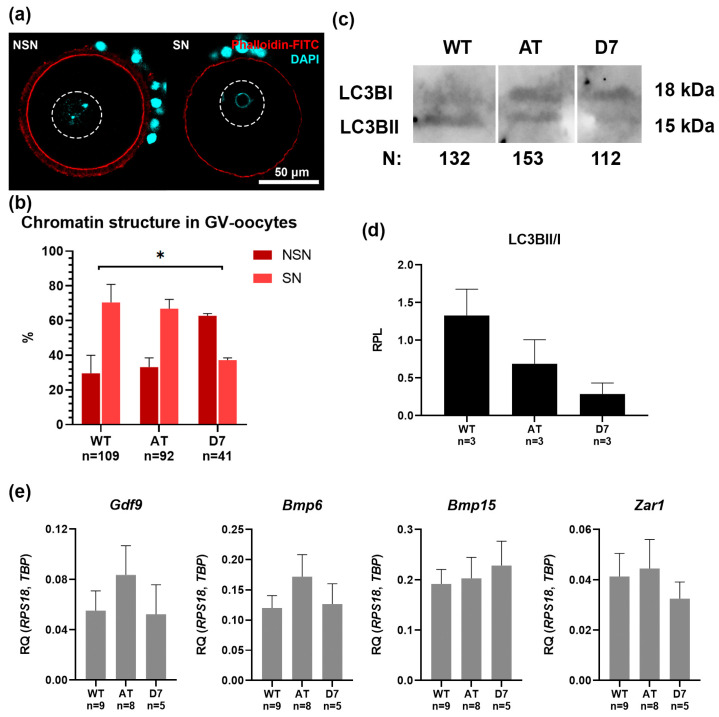
Analysis of GV-oocytes from control (WT) and mutant (AT and D7) female mice. (**a**) GV-oocytes stained with DAPI (cyan) for visualization of SN (surrounded nucleolus) and NSN (non-surrounded nucleolus) chromatin configurations. The dashed line indicates the boundaries of the germinal vesicle. Oocyte boundaries are delineated by phalloidin staining (red). (**b**) Analysis of the ratio of SN and NSN oocytes obtained from ovaries of females induced by HyperOva^®^, M ± SEM, n—the number of oocytes analyzed per group; * *p* < 0.05 according to Two-way ANOVA with Dunnett’s multiple comparisons test. (**c**) Western blot analysis of LC3B protein expression in GV-oocytes obtained from the ovaries of females induced by HyperOva^®^. n—the number of oocytes pooled into a sample for analysis. (**d**) Quantification of LC3B-II/I ratio, M ± SEM, n—the number of samples analyzed per group. (**e**) RT-qPCR analysis of mRNA expression of oocyte-derived growth factors *Gdf9*, *Bmp6*, and *Bmp15*, and the cytoplasmic maturity marker *Zar1*. M ± SEM, n—the number of animals analyzed per group.

To assess the impact of mTERP expression on oocyte maturation, we analyzed chromatin configuration in germinal vesicle (GV)-stage oocytes isolated after hormonal stimulation (Figure 4a). The percentage of oocytes reaching the mature “surrounded nucleolus” (SN) configuration was significantly reduced in the D7 mutant line (37.2%) compared to both the AT mutant (66.9%) and wild-type (WT) controls (70.4%; Figure 4b), indicating a delay in cytoplasmic maturation.

We next measured autophagy levels by Western blot analysis of the LC3B marker (Figure 4c). The LC3B-II/LC3B-I ratio, an indicator of autophagic activity, was significantly reduced in D7 oocytes (1.1) and slightly decreased in AT oocytes (1.4) relative to WT controls (1.7; Figure 4d). These results demonstrate that mTERP mutations, particularly the D7 deletion, disrupt normal autophagy in oocytes.

We also analyzed the expression of key oocyte-specific factors by quantitative RT-PCR (Figure 4e). In the AT mutant group, the growth factors *Gdf9*, *Bmp15*, and *Bmp6* all showed a non-significant trend towards increased expression (*p* > 0.05, t-test). Conversely, *Zar1* expression in the D7 mutant group was reduced by approximately 50% compared to controls, approaching statistical significance (*p* = 0.06, t-test).

Taken together, these findings demonstrate that mTERP mutations, especially the D7 deletion, disrupt critical aspects of oocyte maturation, including chromatin condensation, autophagy, and the expression of key developmental genes.

### 3.4. Quantitative Assessment of Ovarian Reserve Size in Newborn Female Ovaries (4 dpp)

**Figure 5:**
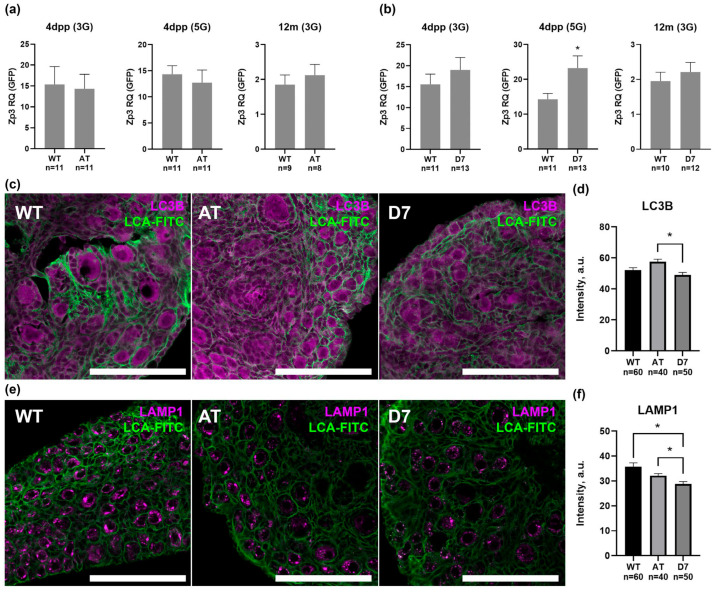
Assessment of ovarian reserve and autophagy levels in the ovaries of control (WT) and mutant (AT and D7) female mice. (**a**,**b**) RT-qPCR analysis of *Zp3* mRNA expression normalized to an external RNA standard (*GFP*) in ovaries of wild-type control (WT) and AT (**a**) and D7 (**b**) mutant female mice at 4 days post-partum (4 dpp) (3G–third generation, 5G–fifth generation of transgenic mice) and 12 months (12 m), M ± SEM, n—the number of animals analyzed per group; * *p* < 0.05 according to Unpaired t-test. (**c**,**e**) Immunohistochemical detection of the autophagy marker LC3B (**c**) and the lysosomal protein LAMP1 (**e**) in ovaries of newborn females. Follicle boundaries are delineated by LCA-FITC staining (green). Scale bar: 100 μm. (**d**,**f**) Analysis of LC3B (**d**) and LAMP1 (**f**) immunofluorescence intensity in ovaries of newborn females in arbitrary signal intensity units (a.u.). M ± SEM, n—the number of ROI analyzed per group, * *p* < 0.05 according to Kruskal–Wallis test with Dunn’s multiple comparisons test.

We next evaluated the impact of mTERP mutations on the initial ovarian reserve by quantifying *Zp3* mRNA levels in the ovaries of newborn mice (4 dpp) from the 3rd and 5th generations (Figure 5). While only minor, non-significant alterations in ovarian reserve were observed in the 3rd generation mice at any age, a pronounced effect emerged by the 5th generation. In these newborns, the ovarian reserve in the D7 group was significantly increased, reaching 162.3% of the wild-type (WT) control level (*p* < 0.05), whereas the AT group remained comparable to WT (88.8%; Figure 5b).

To determine if this altered ovarian reserve correlated with changes in autophagy, we analyzed the expression of the autophagy marker LC3B and the lysosomal marker LAMP1 via immunohistochemistry (Figure 5c,e). Both markers are localized predominantly to the oocyte cytoplasm. Quantitative analysis revealed that the expression of both LC3B and LAMP1 was reduced in the ovaries of D7 newborns (to 90.0% and 80.7% of WT levels, respectively). In contrast, the AT group showed only minor fluctuations in these markers (Figure 5d,f).

Collectively, these data demonstrate that the D7 deletion is associated with both a disruption of autophagy and an unexpected increase in the initial ovarian follicle pool in newborn mice.

## 4. Discussion

This study reveals a critical, non-canonical role for the telomerase-associated protein TERP in mammalian oogenesis. Our findings demonstrate that precise TERP expression is essential for oocyte quality, as both gain- and loss-of-function mutations lead to profound defects. These include compromised meiotic spindle architecture, disrupted cytoplasmic maturation, and altered establishment of the ovarian reserve. We propose that impaired cellular autophagy is the central mechanism mediating these diverse phenotypes.

### 4.1. TERP Dysregulation Compromises Meiotic Spindle Integrity

A central finding of this work is the high frequency of meiotic spindle abnormalities in oocytes from both AT and D7 mutant lines. A properly assembled spindle is essential for accurate chromosome segregation; its failure is a leading cause of aneuploidy, infertility, and developmental disorders [2,25]. The significantly larger spindles in both mutant groups point to a failure in the regulatory mechanisms that control spindle assembly and size. This defect was frequently accompanied by ectopic microtubule-organizing centers. The failure to consolidate MTOCs into two focused spindle poles is a clear sign of aberrant maturation [26,27] and indicates a deeper cellular dysregulation, which our data connect to autophagy.

### 4.2. Impaired Autophagy as the Core Cellular Defect

Our results strongly implicate autophagy as the key cellular process disrupted by TERP mutations. Autophagy is a fundamental pathway that remodels the oocyte cytoplasm and eliminates damaged organelles to ensure developmental competence [6]. In the loss-of-function D7 line, we observed a marked reduction in the LC3B-II/I ratio, a reliable marker of autophagic activity [28]. This correlated with a severe block in cytoplasmic maturation, where few oocytes reached the critical SN chromatin configuration. This link is paramount, as impaired autophagy is known to increase DNA damage and aneuploidy rates [29]. Therefore, we propose that compromised autophagy in D7 oocytes is the primary mechanism underlying the observed spindle defects and subsequent threat to genomic stability.

### 4.3. A Novel Role for TERP in Establishing the Ovarian Reserve

Our study also demonstrates that TERP’s influence extends to the very foundation of female fertility: the establishment of the primordial follicle pool. Perinatal autophagy is instrumental in culling germ cells to define the initial ovarian reserve size [5]. Consistent with this, the loss of TERP function in newborn D7 females correlated with reduced autophagy markers and a paradoxical increase in the ovarian reserve. Of note, this effect on the initial reserve appeared to become more pronounced over successive generations. We hypothesize this reflects a cumulative, heritable impact of the impaired autophagic culling mechanism, though confirming this would require dedicated multi-generational studies incorporating analyses of apoptosis and atresia. However, this numerical advantage proved fleeting. The accelerated depletion of the follicle pool by 12 months of age indicates these supernumerary follicles were of inferior quality and could not extend the reproductive lifespan.

These findings position the TERP as a novel component within the complex regulatory network governing the ovarian reserve, acting alongside well-established factors like the anti-Müllerian hormone (AMH). AMH, secreted by somatic granulosa cells, provides a crucial postnatal regulatory signal. It classically acts as a “brake” on follicle recruitment and, as recent evidence suggests, also actively promotes follicle survival by inducing autophagy [30]. Complementing this somatic, postnatal regulation, our findings demonstrate that TERP operates as an oocyte-intrinsic factor during the perinatal period. Thus, the TERP-autophagy axis represents a foundational, quality-control mechanism that precedes AMH’s later role in postnatal maintenance. The trend towards decreased *Zar1* expression in D7 oocytes further supports the conclusion of poor oocyte quality, as ZAR1 is essential for the oocyte-to-embryo transition [31].

### 4.4. Limitations and Future Directions

We acknowledge that this study, as a foundational characterization, has limitations that highlight clear avenues for future research. Our work establishes a strong correlation between TERP and autophagy, but the precise molecular mechanism connecting them remains to be elucidated. Functional assays, such as rescue experiments with TERP overexpression or the use of autophagy modulators, are the critical next step to establish causality.

Our study focused specifically on ovarian biology. While we noted no overt defects in the animals’ general health or fertility, a systematic, long-term investigation is still required to determine the full systemic effects of these mutations on lifespan and overall health. It is plausible that TERP’s function as an autophagy regulator extends to spermatogenesis. This hypothesis is compelling because autophagy is fundamental to male gamete formation, driving the cytoplasmic remodeling required to produce mature spermatozoa [32]. Although our study focused on females, exploring this potential function in male germ cells is a logical next step. Determining if this novel regulatory axis is sex-specific or a general principle of gamete quality control will be a key future question.

In conclusion, our study establishes TERP as a novel regulator of female gamete quality. We provide evidence that this function is mediated through the regulation of perinatal autophagy, a process essential for defining the initial ovarian reserve and ensuring meiotic fidelity. By uncovering this new regulatory axis, our work provides a potential explanation for cases of idiopathic female infertility and provides potential new targets for strategies aimed at preserving or improving oocyte quality.

## Figures and Tables

**Table 1:** Specific Oligonucleotides Used for Real-Time PCR.

Gene Name	NCBI Gene ID	Primers Sequences, 5′→3′		Tm, °C	Product Length, bp	Product Tm, °C
*Bmp6*	12161	F:	ACCGTACTTTGTGGCAGAGC	59.8	174	84.7
		R:	GAAAAGGCAAAGAGCAGAGTTAG	59.5		
*Bmp15*	12155	F:	GAATCTGATGCCTCTTGTCCTT	59.6	200	83.5
		R:	ATGGCATGGTTGGGTGAAT	60.1		
*Gdf9*	14566	F:	GCCTCCCCGACCTTTAGA	59.4	198	85.5
		R:	TGCCTCAGACTCCACATTTTC	59.6		
*GFP*	–	F:	CATGGCCGACAAGCAGAAGAAC	58.4	226	82.7
		R:	GGCGGCGGTCACGAACTC	58.6		
*Rplp0*	11837	F:	ACTGGTCTAGGACCCGAGAAG	59.5	77	80.9
		R:	TCAATGGTGCCTCTGGAGATT	61.4		
*Rps18*	20084	F:	AAGAAAATTCGAGCCCATAGAGG	62.8	138	86.1
		R:	TAACAGCAAAGGCCCAGAGACT	62.6		
*Tbp*	21374	F:	GTAGCGGTGGCGGGTATCT	61.9	120	84.1
		R:	CGTCTTCAATGTTCTGGGTTATCT	61.3		
*Zar1*	317755	F:	GTGATTCGGATGCCCCTCG	65.3	103	85.6
		R:	GGGCAGGCACATCTAGTTCTT	60.5		
*Zp3*	22788	F:	TGCCAGACCCGAACTCCT	60.5	204	86.4
		R:	TAGCTGGCGCGACTTTGA	60.7		

## Data Availability

The original contributions presented in this study are included in the article. Further inquiries can be directed to the corresponding author.

## Supplementary Materials

The following supporting information can be downloaded at: https://www.mdpi.com/article/10.3390/biomedicines13092166/s1.

## Abbreviations

The following abbreviations are used in this manuscript:

AMPK	AMP-activated protein kinase
AT	Gain-of-function mutant mouse line for TERP
Bmp6	Bone morphogenetic protein 6
Bmp15	Bone morphogenetic protein 15
Cas9	CRISPR-associated protein 9
COCs	Cumulus-oocyte complexes
D7	Loss-of-function mutant mouse line for TERP
DAPI	4′,6-diamidino-2-phenylindole
dPBS	Dulbecco’s Phosphate-Buffered Saline
dpp	Days post-partum
eCG	Equine Chorionic Gonadotropin
FBS	Fetal Bovine Serum
FITC	Fluorescein isothiocyanate
GFP	Green fluorescent protein
GV	Germinal vesicle
HBSS	Hanks’ Balanced Salt Solution
hCG	Human chorionic gonadotropin
hTERC	Human telomerase RNA
ICG SB RAS	Federal Research Center Institute of Cytology and Genetics, Siberian Branch, Russian Academy of Sciences
LAMP1	Lysosomal-associated membrane protein 1
LCA	Lens culinaris agglutinin
LC3B	Microtubule-associated protein 1A/1B-light chain 3B
MII	Metaphase II
MTOCs	Microtubule-organizing centers
NCBI	National Center for Biotechnology Information
NSN	Non-surrounded nucleolus
nt	Nucleotide
O.C.T.	Optimal Cutting Temperature
ORFs	Open reading frames
PBS	Phosphate-buffered saline
PBST	Phosphate-buffered saline with Triton X-100
PCR	Polymerase chain reaction
qRT-PCR	Quantitative reverse transcription polymerase chain reaction
RIPA	Radioimmunoprecipitation assay
ROI	Region of Interest
Rps18	Ribosomal protein S18
RT-qPCR	Real-Time quantitative polymerase chain reaction
SDS	Sodium dodecyl sulfate
SDS-PAGE	Sodium dodecyl-polyacrylamide gel electrophoresis
SEM	Standard Error of Mean
SN	Surrounded nucleolus
Tbp	TATA-box binding protein
TERC	Telomerase RNA component
TERP	Telomerase RNA protein
TERT	Telomerase reverse transcriptase
Tris-HCl	Tris(hydroxymethyl)aminomethane hydrochloride
TSC2	Tuberous sclerosis complex 2
WT	Wild-type
Zar1	Zygote arrest 1
Zp3	Zona pellucida glycoprotein 3
