# Supplementary material for: The Telomerase RNA Protein TERP Exerts a New Function in Safeguarding Female Gamete Quality
Source: Biomedicines. 2025 Sep 5;13(9):2166. doi: 10.3390/biomedicines13092166 (PMC12467170; doi:10.3390/biomedicines13092166)
Supplement: Supplementary file 1 [file biomedicines-13-02166-s001.zip › biomedicines-3834176 Supplementary Table S3.pdf]

Supplementary Table S3. Reproductive performance of WT, AT, and D7 mice.
Breeding trials were conducted over a 6-month period to assess the fecundity of female mice from wild-type (WT), AT (gain-of-function), and D7 (loss-of-function) lines. Data on litter size and sex ratio are summarized below.

**Parameter**	**WT (Control)**	**AT**	**D7**
Breeding scheme (male × female)	1 × 3	1 × 2	1 × 3
Total litters observed	6	6	6
Total pups born	52	31	35
Average litter size (pups/litter)[1]	**8.7**	**5.2**	**5.8**
Cumulative pups per female (6-month period)[2]	17	16	12
Sex ratio (% male)	44%	53%	43%
Sex ratio (% female)	56%	47%	57%

[1] Average litter size is calculated as Total pups born / Total litters observed.

[2] Cumulative pups per female is calculated as Total pups born / number of females in the breeding group. This parameter reflects the overall reproductive output per female over the entire observation period.
