# Supplementary material for: The Telomerase RNA Protein TERP Exerts a New Function in Safeguarding Female Gamete Quality
Source: Biomedicines. 2025 Sep 5;13(9):2166. doi: 10.3390/biomedicines13092166 (PMC12467170; doi:10.3390/biomedicines13092166)
Supplement: Supplementary file 1 [file biomedicines-13-02166-s001.zip › biomedicines-3834176 Supplementary Methods.pdf]

Supplementary Methods

Sequence of the ssDNA template

>

GTATTTAAGGTCGAGGGCGGCTAGGCCTCGGCACCTAACCCTGATTTTCATTAGATG
TGGGTTCTGGTCTTTTGTTCTCCGCCCGCTGTTTTTCTCGCTGACTTCCAGCGGGC
CAGGAAAGTCCAGACCTGCAGCGGGCCACCGCGTTCCCGAGCCTCAAAAACA
AACGTCAGCGCAGG
