# Supplementary material for: The Telomerase RNA Protein TERP Exerts a New Function in Safeguarding Female Gamete Quality
Source: Biomedicines. 2025 Sep 5;13(9):2166. doi: 10.3390/biomedicines13092166 (PMC12467170; doi:10.3390/biomedicines13092166)
Supplement: Supplementary file 1 [file biomedicines-13-02166-s001.zip › biomedicines-3834176 Supplementary Table S1.pdf]

Supplementary Table S1: List of antibodies and key reagents for immunofluorescence and Western Blotting.

Reagent / Antibody	Manufacturer	Catalogue number
**Primary Antibodies**		
Mouse anti-β-tubulin	ABClonal, Wuhan, China	AC021
Rabbit anti-LC3B	ABClonal, Wuhan, China	A11282
Rat anti-LAMP1	Thermo Fisher Scientific, USA	14-1071-82
Rabbit anti-LC3B	ABclonal, Wuhan, China	A11282
**Secondary Antibodies**		
Goat anti-mouse IgG, CF 568	Merck KGaA, Germany	SAB4600312
Goat anti-rabbit IgG, CF 568	Merck KGaA, Germany	SAB4600076
Donkey anti-rat IgG, CF 633	Merck KGaA, Germany	SAB4600133
HRP-conjugated goat anti-rabbit IgG	Jackson ImmunoResearch Labs, USA	111-035-144
**Other Reagents**		
DAPI	Merck KGaA, Germany	268298
Tissue-Tek® O.C.T.™ Compound	Sakura Finetek, Japan	4583
FITC-Lens Culinaris Agglutinin	Thermo Fisher Scientific, USA	L32475
Mowiol	Merck KGaA, Germany	324590
Protease/Phosphatase Inhibitor Cocktail (100X)	Cell Signaling Technology, Inc., USA	5872S
