# Supplementary material for: The Telomerase RNA Protein TERP Exerts a New Function in Safeguarding Female Gamete Quality
Source: Biomedicines. 2025 Sep 5;13(9):2166. doi: 10.3390/biomedicines13092166 (PMC12467170; doi:10.3390/biomedicines13092166)
Supplement: Supplementary file 1 [file biomedicines-13-02166-s001.zip › biomedicines-3834176 Supplementary Table S2.pdf]

Supplementary Table S2. One-year survival and health monitoring of female mice. A cohort of female mice from each line was monitored for general health and survival over a 12-month period starting from one month of age.

**Parameter**	**WT (Control)**	**AT**	**D7**
Initial number of females	6	6	6
Surviving females at 12 months	6	6	5
Survival rate (%)	100%	100%	83.3%
General health observations[1]	No overt pathologies or signs of distress observed.	No overt pathologies or signs of distress observed.	No overt pathologies or signs of distress observed. One female died at 11 months of age from an undetermined cause.

[1] Mice were monitored for overt signs of malformations, pathologies, or distress throughout the 12-month observation period.
